# Human papillomavirus type 16 (HPV-16) IgG antibody among women of reproductive age presenting at a healthcare facility in Central Nigeria: a pilot study

**DOI:** 10.11604/pamj.2021.40.203.28804

**Published:** 2021-12-06

**Authors:** Moses Peter Adoga, Rine Christopher Reuben, Khadijah Abubakar, Victor Baba Oti, Abigail William Zakka

**Affiliations:** 1 Department of Microbiology, Faculty of Natural and Applied Sciences, Nasarawa State University, Keffi, Nasarawa State, Nigeria,; 2 African Centre for Infection and Biomolecular Sciences (ACIBS), Nasarawa State University, Keffi, Nasarawa State, Nigeria,; 3 German Centre for Integrative Biodiversity Research, Halle-Jena-Leipzig, University of Leipzig, Leipzig, Germany,; 4 Department of Microbiology, Kaduna State University, Kaduna State, Nigeria

**Keywords:** Human papillomavirus, IgG antibody, humoral immunity, sexually transmitted disease, epidemiology

## Abstract

Approximately, 70% of cervical cancer cases worldwide are attributable to HPV-16 and HPV-18, with HPV-associated cancers being the second most common infection-related cancers globally. However, there´s paucity of data about this infective agent in Central Nigeria. In a cross-sectional study, we evaluated the seroprevalence of HPV-16 immunoglobulin G (IgG) and risk determinants among women in Central Nigeria as a first step towards evaluating anti-HPV IgM antibody for active cases and determining incidence. Blood samples were collected between August 2016 and January 2018, from 400 consenting women of childbearing age (15-49 years) who completed structured questionnaires. Samples were analyzed using HPV-16 specific IgG ELISA kits (Cusabio Co. Ltd, Germany). Statistical analysis was performed to determine predictors. Overall, we found that 128 (32.0%) had IgG antibody against HPV-16. Seroprevalence by age was 50.0% (15-19 years), 55.0% (20-24 years), 12.9% (25-29 years), 50.0% (30-34 years), 32.1% (35-39 years), 18.2% (40-44 years) and 19.4% (45-49 years) respectively. Factors associated with infection were age (P=0.0002; 95% CI 5.06-31.51), occupation (P<0.0001; 95% CI 1.4-12.6), number of sex partners (P=0.0037; 95% CI 1.27-49.93), history of genital warts (P=0.0203; 95% CI 1.34-9.55) and education level (P<0.0001; 95% CI 3.89-60.11). In addition, forty six (11.5%) reported having the history of genital warts with 268 (67.0%) and 132 (33.0%) subjects being married and single respectively. Individuals who were either artisans or civil servants were 260 (65.0%), whereas 140 (35.0%) were students. Majority, 324 (81.0%), had either primary, secondary or tertiary education with 76 (19.0%) of the subjects having no formal education. In respect of sexual behaviour, 196 (49.0%) reported having at least two sexual partners, out of which 64 (16.0%) had three or more. These findings provide high serological evidence of exposure to HPV-16 in Central Nigeria with implications for national and regional intervention initiatives.

## Introduction

Limited data on human papillomavirus (HPV) is available in Nasarawa State and Central Nigeria in spite of the critical role this virus plays in cancer. Therefore, in the present pilot study which will lead to a more comprehensive investigation, including evaluating anti-HPV IgM antibody for active cases and determining incidence of infection, we found that the prevalence of IgG antibody to HPV-16 among women of child-bearing age who presented at a healthcare facility in Central Nigeria was 32.0%.

HPV is a small non-enveloped icosahedral deoxyribonucleic acid (DNA) virus that can replicate in the nucleus of squamous epithelial cells, and infection with the virus is a major risk factor for squamous intraepithelial lesions and cervical cancer [1]. The virus mostly invades the genital areas of men and women, including the skin of the penis, vulva, anus, the linings of the vagina, cervix, and rectum [2].

Globally, approximately 70% of all cases of cervical cancer are attributable to HPV-16 and HPV-18; and cervical cancer is the fourth most frequent cancer among women worldwide [3,4]. In Nigeria, a substantial burden of cancer among women are those associated with HPV; and the second most common infection-related cancers worldwide are those attributable to the virus [5]. Over 120 types of HPV have been identified to date, and fifteen (types 16, 18, 31, 33, 35, 39, 45, 51, 52, 56, 58, 59, 68, 73, and 82) have been classified as high-risk types or carcinogenic, with HPV-16 being the most frequently isolated among cervical cancer patients [6-8].

Moreover, about 80% of HPV-attributable cancer cases occur in developing countries, with sub-Saharan Africa, Melanesia, Latin America and the Caribbean, south-central and south-east Asia having the highest incident cases [9]. Several major risk factors for cervical cancer are common in sub-Saharan African countries, including prolonged HPV infections and HIV/AIDS, which is endemic in this region [10].

Notwithstanding the critical role of HPV in cancers, especially those of the cervix, there is a paucity of published data on HPV infection and risk determinants among women in Central Nigeria in spite of the public health significance of such evidence. Therefore, in the present pilot study, we evaluated exposure to HPV-16 by determining the prevalence of HPV-16 specific immunoglobulin G (IgG) antibody. We further assessed risk determinants among women of child-bearing age who presented at a healthcare facility in Central Nigeria. This allows for a more comprehensive study that will involve identifying the proportion of women with active cases (IgM antibody) and as well evaluating the incidence of infection to determine the level of risk posed to the population. As with other developing countries, such information is critical for intervention initiatives in Nigeria where knowledge of prevention initiatives and prophylactic vaccine availability have been found to be low among women [11].

## Methods

**Study area:** the healthcare facility where the study participants were recruited was located in Keffi Town, Nasarawa State. Keffi is approximately 68 km away from Abuja, the capital city of Nigeria. Keffi is located between latitude 8 5´N and longitude 7 8´E and situated on an altitude of 850 m above sea level [12].

**Ethical approval and informed consent:** prior to the study, ethical clearance (ref: FMC/KF/HREC/098/16) was obtained from the Health Research Ethics Committee (Reg. No. NHREC/21/12/2012) at the Federal Medical Centre, Keffi, Nigeria, in line with the national code of ethics for biomedical research involving human subjects and the Helsinki Declaration. Informed consent was also obtained from each participant before enrollment. This was done by ensuring that each subject read and signed a consent form or had a translator read it for them before appending their signatures.

**Study population:** we arrived at a representative sample size using the formula propounded by Naing as previously described [13]. The study participants consisted of eligible 400 consenting women of childbearing age (15-49 years) presenting at Riyan Clinic, Keffi, beginning from August 2016 to January 2018. Sociodemographic information of the participants was obtained by a trained assistant in a structured questionnaire. Such information included; age, occupation, history of genital warts, marital status, educational level and number of sexual partners. Each subject self-administered a questionnaire or had the assistant read and translate it to them before completion.

**Exclusion and inclusion criteria:** women who had history of HPV vaccination were excluded from the study. This ensured that only individuals who had anti-HPV-16 IgG antibody due to natural infection were included in the study.

**Sample collection:** 5 ml of blood was collected aseptically from each participant by venipuncture into a sterile plain universal container. The blood was allowed to clot for 30 minutes and centrifuged at 3,000 rpm for 20 minutes. A Pasteur pipette was used to harvest and dispense each serum into a separate, properly labeled sample vial and stored at -20° C until use.

**Anti-HPV-16 IgG antibodies detection:** the HPV-16 specific IgG ELISA Kits (Cusabio Co Ltd, Germany) were used to detect anti-HPV IgG antibody in the sera of subjects. The test procedure and result interpretation were carried out according to the manufacturer´s protocol. Included in the kits are negative and positive controls; and microtiter plates pre-coated with HPV-16 antigens. After the addition of uniformly diluted sera to the microtiter wells and 30-minute incubation at 37°C, wells were washed. This was followed by adding horseradish peroxidase (HRP)-conjugated anti-human IgG and a 30-minute incubation at 37°C. It was then washed thoroughly. The addition of tetramethylbenzidine (TMB) substrate solution to each well was done and termination of the enzyme-substrate reaction was effected by adding sulphuric acid solution. A microplate reader (Tecan Infinite m200 Pro, Tecan Group Ltd., Mannedorf) was used at 450 nm to spectrometrically measure the intensity of colour changes. Manufacturer´s protocol was then used to determine, through optical density, the valence of anti-HPV-16 IgG antibody and the cut-off value for a positive HPV-16 result.

**Statistical analysis:** descriptive statistical analysis was performed on data using the Smith´s Statistical Package Version 2.80 (Claremont, California, USA) and Chi-squares (χ^2^) were calculated. The p-values and 95% confidence interval (CI) were determined for each of the variables (age, marital status, level of education, occupation, number of sexual partners, and history of genital warts). Associations between sero-positivity for HPV-16 IgG antibodies and the tested variables were evaluated using Chi-square tests. An association was considered statistically significant when p ≤ 0.05.

## Results

A total of 400 women of child-bearing age with a mean age of 35.2 years voluntarily participated in this study. Overall, 128 (32.0%) had IgG antibody against HPV-16. Seroprevalence in relation to age was 50.0% (15-19 years), 55.0% (20-24 years), 12.9% (25-29 years), 50.0% (30-34 years), 32.1% (35-39 years), 18.2% (40-44 years) and 19.4% (45-49 years) respectively. Table 1 shows factors associated with infection such as age (P=0.0002; 95% CI 5.06-31.51), occupation (P < 0.0001; 95% CI 1.4-12.6), number of sex partners (P=0.0037; 95% CI 1.27-49.93), history of genital warts (P=0.0203; 95% CI 1.34-9.55) and education level (P < 0.0001; 95% CI 3.89-60.11).

**Table 1:** association of serological evidence of exposure to HPV-16 with sociodemographic characteristics among women of reproductive age at a healthcare facility in Central Nigeria

Variable	No. screened	No. positive	Prevalence (%)	p-value (95% CI)
**Age (years)**				
15-19	4	2	50.0	
20-24	80	44	55.0	
25-29	62	8	12.9	
30-34	60	30	50.0	0.0002 (5.06-31.51)
35-39	56	18	32.1	
40-44	66	12	18.2	
45-49	72	14	19.4	
**Marital status**				
Married	268	80	29.9	0.3498 (13.9-67.8)
single	132	48	36.4	
**Occupation**				
Artisans	184	4	2.2	
Civil servants	76	58	76.3	< 0.0001 (1.4-12.6)
Students	140	66	47.1	
**Education level**				
Secondary	80	20	25.0	
Primary	40	14	35.0	<0.0001 (3.89-60.11)
Uneducated	76	50	65.8	
Tertiary	204	44	21.6	
**Sexual partners**				
0	20	0	0.0	
1	184	41	22.3	
2	132	49	37.1	0.0037 (1.27-49.93)
3	36	17	47.2	
≥ 4	28	21	75.0	
**History of genital warts**				
Yes	46	25	54.3	0.0203 (1.34-9.55)
No	354	103	29.1	

In addition, forty-six (11.5%) reported having the history of genital warts with 268 (67.0%) and 132 (33.0%) subjects being married and single respectively. Individuals who were either artisans or civil servants were 260 (65.0%), whereas 140 (35.0%) were students. Majority, 324 (81.0%), had either primary, secondary or tertiary education with 76 (19.0%) of the subjects having no formal education. In respect of sexual behaviour, 196 (49.0%) reported having at least two sexual partners, out of which 64 (16.0%) had three or more (Table 1).

## Discussion

In this study, we found high serological evidence of exposure to HPV-16 to be 32.0% by determining the proportion of individuals who had HPV-16 specific IgG antibody among women of reproductive age who presented at a healthcare facility in Central Nigeria. However, this does not represent the total burden of infection since a study to identify active (IgM antibody) and incident cases is underway. Nevertheless, the evidence of exposure recorded in this study, defined as being HPV-16 IgG antibody positive, is high in comparison to similar studies carried out in certain parts of Southern Nigeria. For instance, it was 23.6% in Osogbo and 10% in Port Harcourt [11,14]. Moreover, the burden of the infection doesn´t quite look dissimilar with reports from other African countries. For example, studies in Burkina Faso and Rwanda found 38.3% and 5.9% respectively [1,15].

The distribution of HPV infection according to age appeared to be high in all age groups and there was a statistically significant association between age and infection (p < 0.05). The high prevalence in all age groups agrees with a previous study in Southern Nigeria [16], and underscores the potential risk this virus poses to the entire population and the need for comprehensive intervention initiatives. Additionally, exposure to the virus was not significantly associated with marital status even though evidence of exposure to the virus was slightly higher among individuals who were single with 36.4% seroprevalence than the married subjects who had a seroprevalence of 29.9%. Similarly, a study in Rwanda reported a higher prevalence among single than married women [15]. Since transmission is primarily through sexual contact, this might find explanation in the fact that single women are obviously more likely to keep multiple sexual partners than married ones; just as confirmed in the present study that the number of sexual partners is a predictor of infection.

With respect to occupation, anti-HPV-16 IgG antibody prevalence was 76.3%, 47.1% and 2.2% among civil servants, students and artisans respectively; with this variable being a factor associated with infection (p < 0.05). Moreover, our findings revealed a statistically significant higher prevalence among uneducated subjects and those with only primary education. The fact that these two groups of individuals lack basic education that guarantees access to prevention information resources may be a suitable explanation for this observation. There was a statistically significant association between the number of sexual partners and the risk of exposure (p < 0.05). This was no surprise in view of the relationship between HPV and sexual activities. Moreover, our findings show significant relationship (p < 0.05) between the history of genital warts among subjects with sero-positivity of HPV-16 IgG antibodies. This further confirms the association of HPV with genital warts which typically occurs on the vulva, cervix, vagina and around the anus of infected women.

## Conclusion

This study demonstrates high serological evidence of exposure to HPV-16 among women of reproductive age within the study area; and the circulation of this virus within the population portends grave public health consequences. Importantly, with growing scientific evidence linking HPV to cervical and anogenital cancers (involving the anus, penis, vagina and vulva) as well as some head and neck cancers, this study undoubtedly provides critical information that will inform cancer prevention policy. Findings in this pilot study provide critical insights into the burden of HPV infection in a healthcare setting in Central Nigeria with implication for national and regional intervention initiatives, including prophylactic vaccination and other prevention services.

### What is known about this topic

- HPV-16 and HPV-18 are associated with about 70% of cervical cancer cases globally, and the second most common infection-related cancers are HPV-associated;
- Among women worldwide, cervical cancer is the fourth most frequent cancer and much of the burden of cancer among women in Nigeria are attributable to HPV;
- HPV-16 is the most frequently isolated among cervical cancer patients out of the over 120 types of HPV that have been identified to date.

### What this study adds

- This study demonstrates high serological evidence of exposure to HPV in the study population and provides the basis for evaluation of the incidence of infection;
- Insights into the burden of HPV infection in the study area is provided to inform local and regional intervention initiatives;
- It provides baseline data that can inform further studies.
